# Prevalence and Pattern of Pain Presentation among Patients Attending a Tertiary Dental Center in a Southern Region of Nigeria

**DOI:** 10.5681/joddd.2010.012

**Published:** 2010-06-24

**Authors:** Olufemi Gbenga Omitola, Abiodun Olabisi Arigbede

**Affiliations:** ^1^ Lecturer, Department of Oral Pathology and Biology, University of Port Harcourt, Rivers State, Nigeria; ^2^ Senior Lecturer, Department of Restorative Dentistry, University of Port Harcourt, Rivers State, Nigeria

**Keywords:** Orofacial pain, pattern, prevalence, response

## Abstract

**Background and aims:**

Orofacial pain is one of the most common reasons for attendance at the dental clinic. The prevalence and the sources of orofacial pain vary from place to place and patients’ response to pain also differs. The aim of this study was to determine the prevalence and pattern of orofacial pain as well as the factors influencing patients’ response to orofacial pain among patients presenting for dental treatment in our center.

**Materials and methods:**

Consecutive patients presenting with orofacial pain at the Dental Center, University of Port Harcourt Teaching Hospital during the one-year period of the study were recruited into the study. Patients’ demographic data were collected and recorded in special forms. Patients were later examined to confirm the specific cause of pain. This was also recorded. Data generated were analyzed using SPSS for Windows.

**Results:**

Four hundred and forty-eight patients out of the 2,426 patients seen during the study period had orofacial pain, yielding a prevalence rate of 18.5%. There were 255 females and 193 males with most patients in the 17-27-year age group (49.1%). Lower jaw was commonly affected. Most patients presented after a period of at least one month, with severe and spontaneous pain. Most patients had drug therapy before presentation. Caries was the most common source of orofacial pain.

**Conclusion:**

Orofacial pain was not uncommon in our center and it accounted for about 20% of patients’ attendance in the center. Dental caries and periodontal diseases were the most common sources of orofacial pain while temporomandibu-lar joint pain and atypical facial pain were not common in our center. Young adults and females were most commonly affected.

## Introduction

Pain is an unpleasant subjective sensation connected with a potentially damaged tissue.^1^ Orofacial pain is a broad concept, consisting of facial, oral or referred pain. Facial pain occurs in the area under the orbitomental line, above the neck and in front of the ear, while oral pain occurs in the area of the oral cavity.^2^ Oral pain might arise from the tongue, oral mucosa, maxillary bone, mandibular bone and it might be dental in origin. Dental pain can arise from the tooth itself or from its supporting structures and can be acute or chronic with different characteristics.^1^

Pain is a very frequent reason for patients’ attendance at the dental clinic and most of the pain is dental in origin.^1 , 3 , 4^ Dental pain has been shown to influence patients in different ways.^1 - 3 , 5 , 6^ The most common influences are: consulting a dentist or doctor; avoiding certain foods; taking medications and disruption of sleep.^1 , 3^ Several behavioral influences, including reduced social contacts and inability to work, have also been reported.^5 , 6^

Patients’ response to pain perception has also been shown to vary and depend on several factors like sex, age, previous pain experience etc.^7^ Some of these responses may include care seeking and self drug therapy.,^10^

Several studies are available on the prevalence, pattern and impact of orofacial pain on the patients.^11 - 1 , 4^ However, there is none from this area of the country. The present study was thus designed with the aim of determining the prevalence and pattern of orofacial pain as well as the factors influencing patients’ response to orofacial pain among patients presenting for dental treatment in our center. This information is vital for designing oral health care preventive and management strategies.

## Materials and methods

This is a one-year hospital-based cross-sectional study of patients presenting with orofacial pain in the Dental Center of University of Port Harcourt Teaching Hospital, Port Harcourt, Nigeria. The hospital is a major referral center in the southern region of Nigeria. The Dental center of the hospital became a tertiary center about 5 years ago.

Patients presenting with oral and/or facial pain during the period of the study were recruited after they had accepted to participate in the study. Consecutive pediatric and adult (male and female) patients with primary or referred orofacial pain were included. Patients whose presenting complaints and clinical findings did not include orofacial pain were excluded.

The bio-data of the patients and the clinical history of the pain were collected and recorded in special forms. The data collected included: age, sex, marital status, occupation, the location of the pain, duration and characteristics of the pain. History of drug intake in response to the pain and associated symptoms such as headache and sleep disturbance were included. The patients were later examined to ascertain the specific causes of pain; these were also documented in the special forms. The history taking and examination were carried out by the same examiner for all the patients. Patients’ confidentiality and anonymity were preserved.

Data were analyzed using SPSS for Windows, version 12.0 (SPSS Inc, Chicago Illinois, USA). Summary statistics (frequency, percentage) were performed to compare prevalence and pattern of presentation. Chi-square test was used to assess the relationship between pathology causing pain and the demographic variables (i.e. age and gender).

## Results

**Figure 1:**
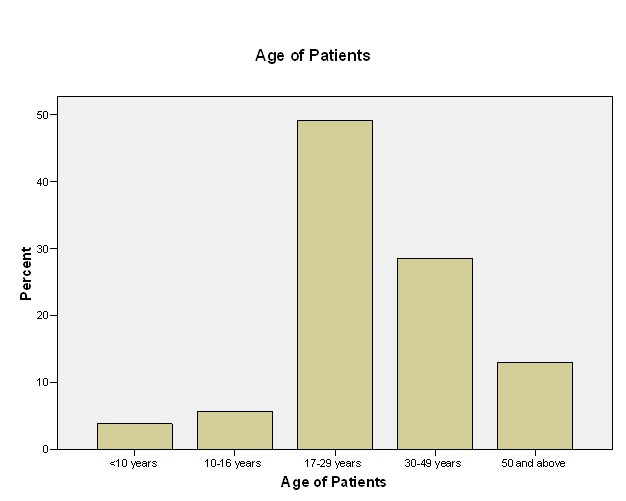
Age distribution of patients with oro-facial pain.

A total of 2,426 patients were seen during the one-year period of the study, out of which 448 patients presented with orofacial pain. Thus, the prevalence of orofacial pain was 18.5%. Among the patients presenting with orofacial pain, 255 (56.9%) were females while the remaining 193 (43.1%) were males. Most of the patients presenting with orofacial pain were within the age range of 17-29 years (49.1%), followed by patients within the age range of 30-49 years (28.6%) (Figure 1). Regarding the marital status, single patients were the largest group accounting for 247 (55.1%) cases, closely followed by the group comprising 191 married individuals (42.6%). The other patients were either divorced (2; 0.4%) or widowed (7; 1.6%).

With respect to the site of pain, lower jaw was the most common site with 218 (48.7%) cases, followed by the upper jaw with 169 (37.7%) cases; 61 (13.6%) cases were located on both the upper and lower jaws. Regarding the side of the face involved, 188 (42%) cases were on the left side and 172 (38.6%) were on the right side; the remaining 86 (19.2%) cases were on both sides.

Most of the patients (179; 40%) presented to the clinic after they had experienced the pain for at least one month; 135 (30.1%) patients presented when the duration of the pain was between one week and a month while only 134 (29.9%) presented to the clinic within one week of experiencing the pain.

Regarding the nature of pain, most (165; 36.8%) of the patients described it as stabbing; 161 (35.9%) stated that the pain they experienced was throbbing while 111 (28.4%) patients said that the pain had a dull nature. In 313 (69.9%) of the cases the pain was spontaneous while in 131 (29.2%) cases the pain was non-spontaneous. Two hundred and eighty-five (63.5%) of the patients had recurrent pain whereas in 157 (35.0%) cases the pain was persistent. Furthermore, 69 (15.4%) patients described their pain as very severe; 261 (58.1%) patients described it as severe; 79 (17.6%) described it as less severe, while the remaining 37 (8.3%) patients described it as not severe.

Regarding drug therapy before presentation, 126 patients were on antibiotics, 33 (26.2%) of which had been prescribed by a qualified health professional. Similarly, 316 patients were on analgesics while only 56 (17.7%) of these had been prescribed. Regarding associated complaints, 277 (61.8%) patients had sleep disturbances while 250 (55.8%) had headaches.

With respect to the source of pain, dental caries involving the pulp was the most common source of orofacial pain (296; 63.5%), followed by periodontal diseases (101; 21.7%). Other sources of oral pain were tooth fracture (46; 9.9%), jaw fracture (8; 1.7%), oral ulcers (3; 0.6%), infected benign tumor (2; 0.4%), malignant tumor (1; 0.2%), temporomandibular joint pain (5; 1.1%) and atypical orofacial pain (4; 0.9%) (Table 10).^1^

Cross-tabulation of age and gender with the sources of pain showed that dental caries and periodontal diseases were more predominant within the 17-29-year age group and among the females. On the other hand, tooth fracture was more predominant within the 30-49-year age group and among the males. Finally, temporomandibular joint pain was more common among the males while atypical orofacial pain was more common among the females (Table 1). However, chi-square test did not reveal any significant differences between the pathologic entity causing the pain and the demographic variables (Table 1).

**Table 1:** Age and gender distribution of sources of pain

			Age (year)			Gender		Total (%)
Condition^*^	1-9 yrs	10-16 yrs	17-29 yrs	30-49 yrs	≥50 yrs	Male	Female	
Caries	16	18	158	85	19	123	173	296 (63.5%)
Periodontal diseases	1	4	47	27	22	33	68	101 (21.7%)
Tooth fracture	0	1	13	17	15	26	20	46 (9.9%)
Jaw fracture	0	1	1	3	3	4	4	8 (1.7%)
Foreign body	0	0	0	0	0	0	0	0 (0%)
Oral ulcer	0	0	1	1	1	1	2	3 (0.6%)
Infected benign tumour	0	0	2	0	0	1	1	2 (0.4%)
Malignant tumour	0	0	1	0	0	1	0	1 (0.2%)
Dental prosthesis	0	0	0	0	0	0	0	0 (0%)
Referred pain	0	0	0	0	0	0	0	0 (0%)
Temporo-mandibular joint pain	0	0	0	2	3	3	2	5 (1.1%)
Atypical orofacial pain	0	0	0	2	2	1	3	4 (0.9%)
Total	17 (3.7%)	24 (5.2%)	223 (47.8%)	137 (29.4%)	65 (13.9%)	193 (41.4%)	273 (58.6%)	466 (100%)

^*^Some patients have more than one source of pain.

## Discussion

The prevalence of orofacial pain in our study was 18.5%. Prevalence of orofacial pain as reported by various researchers varies from place to place and among different age groups.,^11 - 15^ Nomura et al^12^ reported a prevalence of 33.7% among 11-12 year-old school children in Brazil; this is similar to a prevalence of 30.4% reported by Pau et al^15^ among 11-14 years old school children in Pakistan. In their study, Macfarlane el al^13^reported a lower prevalence of 23% among young adults aged 30-31 in Wales, United Kingdom. Among adults, Kikwilu et al^3^ reported a higher prevalence of 58.8% in Tanzania while McMillan et al^11^ in a study on Cantonese-speaking Chinese in Hong Kong reported a prevalence of 41.6%, which dropped to 24.2% when sensitivity was excluded. In a study on adults in Benin City Nigeria, Okunseri el al^14^ reported a prevalence of 34%. Our study investigated the prevalence of oral pain in a population of children and adults, and we reported a prevalence of 18.5% which is slightly higher but similar to 16.49% reported by Ivana et al^1^ in Zagreb among patients the same age.

McMillan et al^11^ in their study reported no gender differences in pain prevalence among their patients. Similarly, in the earlier study conducted at Ile Ife by Fagade et al,^9^ no gender differences in pain perception were reported. However, in the present study and that of Ivana et al,^1^ female predominance was reported. It has been noted that females tend to have a lower threshold of pain and thus tend to seek medical attention much earlier than males who appear to tolerate pain better.^7 , 8^ This might be a reason for this observation. It has also been shown that, generally, females tends to seek medical help more promptly than their male counterparts,^16^ which might be another reason for the female predominance.

In this study, most of the patients with oral pain were within the 17-29-year age bracket. This is not surprising because our study also revealed that caries with pulpal involvement is the most common source of oral pain within this age bracket and more female are affected. These findings are consistent with the earlier reports which suggest higher prevalence of dental caries among young adults and females.^4 , 14 , 17^ The fact that the study was conducted near a tertiary student community could also be responsible for having a higher prevalence of dental pain among young adults. Our report, therefore, may not be a true reflection of what is obtained in the general population.

In addition, dental caries as a major cause of dental pain as reported in our study had earlier been reported by several authors from different parts of Africa.^4 , 14^ In their study in Burkina Faso, Varenne et al^4^ reported that 52.4% of clinic attendance was as a result of pain due to dental caries. Oginni et al^17^ in Ile Ife, Nigeria and van Palenstein et al^18^ in Tanzania reported that the most common reason for dental treatment demand in their center was dental caries and its sequelae. In addition, periodontal diseases are the second most frequent reason why patients present to the clinic on account of oral pain. This is consistent with the report of Varenne et al^4^ in Burkina Faso but differs in the respect that periodontal diseases are more frequent in males, while in this study they were more frequent in females. However, both studies agreed with the fact that dental traumas are more frequent in males.

Analysis of the relationship between the jaws and sides involved showed that oral pain was more commonly located on the lower jaw and on the left side of the face in most cases. The reason is not clear; however, the lower molars are more prone to caries, pericoronitis and perhaps referred pain from the heart.^19 , 20^

Regarding duration before presentation at the clinic, our experience is not different from reports from Nigeria and other parts of Africa,^3 , 14 , 17^ where patients delay their visit to the clinics and only come when the pain becomes unbearable. Most of our patients presented after a duration of at least one month. The reasons most patients gave for this attitude were: lack of funds;^3^ hoping that the pain would disappear on its own without treatment;^3 , 1^ they were on medications which on most time were self-prescribed.,^1^

Even though most patients postponed their visits to the clinic, most of them had pain which was stabbing and throbbing in nature; they also had spontaneous pain which they described as severe. These characteristics are more associated with acute dental pain rather than chronic pain, which is expected, based on the time they present to the clinic. This is explained by the fact that they visited the clinic when there was acute exacerbation of chronic dental pain. This observation is similar to the experience of Ivana et al^1^ in their study where 87.42% of the patients had acute pain while 12.58% had chronic pain.

Most of the patients were on medication before presentation at the clinic. Most disturbing is the fact that about 75% of the patients on antibiotics and about 80% of the patients on analgesics had not been visited by any health professional. The impact of this behavior on emergence of resistant strains of microorganisms and development of irreversible pulpal damage cannot be overemphasized. This lifestyle further corroborates the earlier speculation that patients present late to the clinic because they are engaged in self treatment, hoping that the condition will disappear with time.^3^ The danger of this attitude cannot be overemphasized, especially when more sinister conditions like oral cancers are involved because the mortality and morbidity of most cancers depend on the stage at presentation; patients presenting earlier have a better chance of survival.^2 , 2^

## Conclusion

Orofacial pain is not uncommon in our center and accounts for about a fifth of patients’ attendance in the center. Dental caries and periodontal diseases are the most common sources of orofacial pain while temporomandibular joint pain and atypical facial pain are not common in our center. Young adults within the 17-29-year age group are most commonly affected. Overall, females are affected and the mandible is more involved. Most patients present late to the clinic and only present when the pain becomes unbearable after engaging in self-medication.

It is therefore important to educate patients in this region and other parts of Africa in the dangers associated with self-medication and late presentation for treatment. In addition, the government should address the issue of poverty in this region so that patients can better access available health care facilities because most delays in presentation are due to a lack of funds on behalf of the patients.
